# Progesterone Via its Type-A Receptor Promotes Myometrial Gap Junction Coupling

**DOI:** 10.1038/s41598-017-13488-9

**Published:** 2017-10-17

**Authors:** Lubna Nadeem, Oksana Shynlova, Sam Mesiano, Stephen Lye

**Affiliations:** 1 Lunenfeld Tanenbaum Research Institute, Mount Sinai Hospital, Toronto, Canada; 2 Department of Physiology University of Toronto, Ontario, Canada; 3 Department of Obstetrics & Gynaecology, University of Toronto, Ontario, Canada; 4 Department of Reproductive Biology, Case Western Reserve University, Cleveland, Ohio, USA

## Abstract

Effective labour contractions require synchronization of myometrial cells through gap junctions (GJs). Clasically, progesterone (P4) is known to inhibit the expression of connexin-43 (Cx43, major component of GJs) and GJ formation in myometrium. Our current study is based on a striking observation that challenges this dogma. We observed conspicuous differences in the intracellular localization of Cx43 protein in PRA versus PRB expressing myocytes. Thus in P4 stimulated PRA cells Cx43 protein forms GJs, whereas in PRB cells the forward trafficking of Cx43 and GJ formation is inhibited even when Cx43 is overexpressed. We found that P4, via PRA/B, differentially regulates Cx43 translation to generate a Cx43-20 K isoform, which facilitates the transport of full length Cx43 to plasma membrane. The P4 mediated regulation of Cx43 trafficking and GJ formation occurs via non-genomic pathway and involves the regulation of mTOR signaling since inhibition of this pathway restored the Cx43 trafficking defect in PRB cells. We propose that PRA is a master regulator of Cx43 expression, GJ formation and myocyte connectivity/synchronization for labour.

## Introduction

During most of pregnancy, the uterine muscle (myometrium) is relaxed and in quiescent state to facilitate the growing fetus. However, prior to the onset of labour, the myometrium becomes activated through an increase in the expression of a cassette of contraction-associated proteins including those that control myometrial excitability, especially connexin 43 (Cx43), which forms gap junctions (GJs) between myometrial cells^1,2^. GJ channels facilitate cell-cell communication by coupling cells electrically and metabolically to support the transfer of small metabolites, second messengers and ions^3,4^. Myometrial contractions within the uterus of all species arise from spontaneous action potentials due to transient changes in the plasma membrane (PM) permeability leading to elevation of intracellular Ca^2+^ and the propagation of the action potential between electrically coupled cells. GJ-mediated cell coupling between the uterine myocytes is indispensable to synchronize the labour contractions needed for normal parturition^5–8^.

Connexins are transmembrane proteins that oligomerize and assemble into hemichannels consisting of six molecules. The hemichannels form GJs at the PM when they bind to hemichannels of neighbouring cells^3,9,10^. More than 20 different connexins exist in humans with Cx43 being the predominant form in most tissues^11–14^. It has been established that labour does not occur in the absence of Cx43 containing GJs between myocytes and that the transport of Cx43 to PM is more critical than the increase in Cx43 synthesis alone. Formation of GJs require a sequential series of strictly regulated steps including: 1) Cx43 mRNA transcription^15–17^; 2) translation of Cx43 mRNA in the rough endoplasmic reticulum (ER) to generate full length Cx43 (Cx43-FL) and N-terminally truncated short isoforms of Cx43 (trafficking chaperons for Cx43-FL)^18,19^, 3) forward trafficking of Cx43-FL to the Golgi^20^ and its oligomerization into connexons^18^, 4) actin-mediated transport of connexons to PM^21^, 5) insertion of connexons into PM^20,22^, and 6) assembly of opposing connexons into functional GJs^18,22–24^. The Cx43 mRNA transcript undergoes alternative translation via a cap-independent mechanism to generate at least six N-terminally truncated isoforms (sizes: 32, 29, 26, 20, 11 and 7 KDa), that participate in trafficking of Cx43-FL to the PM^19^. The predominantly expressed short isoform, Cx43-20 K, is restricted to the ER/Golgi network and is not detected at the PM^19^. Cx43-20 K is thought to interact with Cx43-FL and facilitate its ER-to-Golgi transport and hence forward trafficking to PM and GJ formation^19^.

Cx43 synthesis and GJ formation in myometrium is hormonally regulated. Despite the presence of Cx43 protein throughout gestation, GJs appear only prior to labour (both term and preterm) in rodents^16^ and in humans^25^. In lower mammals the expression of Cx43 and GJ formation in myometrium has been positively correlated with plasma estrogen (E2) levels^15,16^ and negatively correlated with P4 treatment^26^. In rodents, the E2/P4 ratio is low during gestation and increases at term due to decreased systemic P4 levels as a result of luteolysis. Correspondingly, the expression of Cx43 protein in rodent gestational tissues is also low during early gestation, increases gradually with pregnancy and reaches maximum levels during labour^16^, mirroring the change in the E2/P4 ratio. P4 via PRs inhibits Cx43 synthesis directly by trans-repressing Cx43 transcription and indirectly by blocking the action of E2 as treatment of ovariectomized rats (which cannot produce P4), with E2 increases Cx43 expression, while administration of P4 blocks E2-induced Cx43 synthesis and trafficking^26,27^. Moreover, in pregnant rats, administration of E2 or premature blockage of the P4 action by RU486 increases Cx43 abundance, GJ formation in the myometrium and promotes preterm labour^15,17,28^. Thus, in rodents myometrial cell Cx43 expression and GJ formation is tightly controlled by E2 and P4 and strongly correlated with transition of the uterus from the relaxed to the labouring state at term and preterm^29^.

In women, circulating E2 and P4 levels are high throughout pregnancy reaching a maximum at term, and parturition occurs without a systemic P4 withdrawal. In this context the hormonal control of myometrial cell Cx43 expression and GJ formation at parturition is thought to be regulated by changes in myometrial cell responsiveness to P4 and E2^30–33^. We have recently provided molecular evidence for a novel mechanism of functional P4 withdrawal as a result of unliganding of nuclear PRA that switches the PRA isoform from a transcriptional repressor to a transcriptional activator of Cx43^34^. We proposed that throughout pregnancy repression of Cx43 transcription by P4 is mediated through its receptor PRB. In the transition towards labour PRA expression is elevated and metabolic inactivation of P4 in myometrial nuclei results in the dominance of unliganded PRA in the nucleus which activates the Cx43 transcription. In addition, we found that P4 binding to PRs differently affects PRs’ cellular localization and function. Typical of most steroid receptors, the unliganded PRB resides in the cytoplasm and translocates to the nucleus upon P4 binding, whereas PRA deviates from this classical behavior in that in its P4-liganded state, PRA resides in the cytoplasm while its unliganded form is predominantly nuclear^34^. The role of cytoplasmic, liganded PRA is unknown. Our current study investigated whether cytoplasmic PRA (liganded to P4) regulated intracellular Cx43 trafficking and GJ formation in human myometrial cells and pregnant human myometrium. We report here that P4 via PRA/B differentially regulates Cx43 trafficking and GJ formation in myometrium such that it inhibits Cx43 trafficking and GJ formation through PRB but promotes it through PRA. Our results suggest that the P4 receptors intricately regulate Cx43 synthesis and trafficking during pregnancy and labour and that receptor PRA is the master regulator of Cx43 synthesis, trafficking and GJ formation during labour.

## Results

### P4-Liganded PRA promotes while P4-Liganded PRB inhibits GJ coupling of uterine myocytes

**Figure 1:**
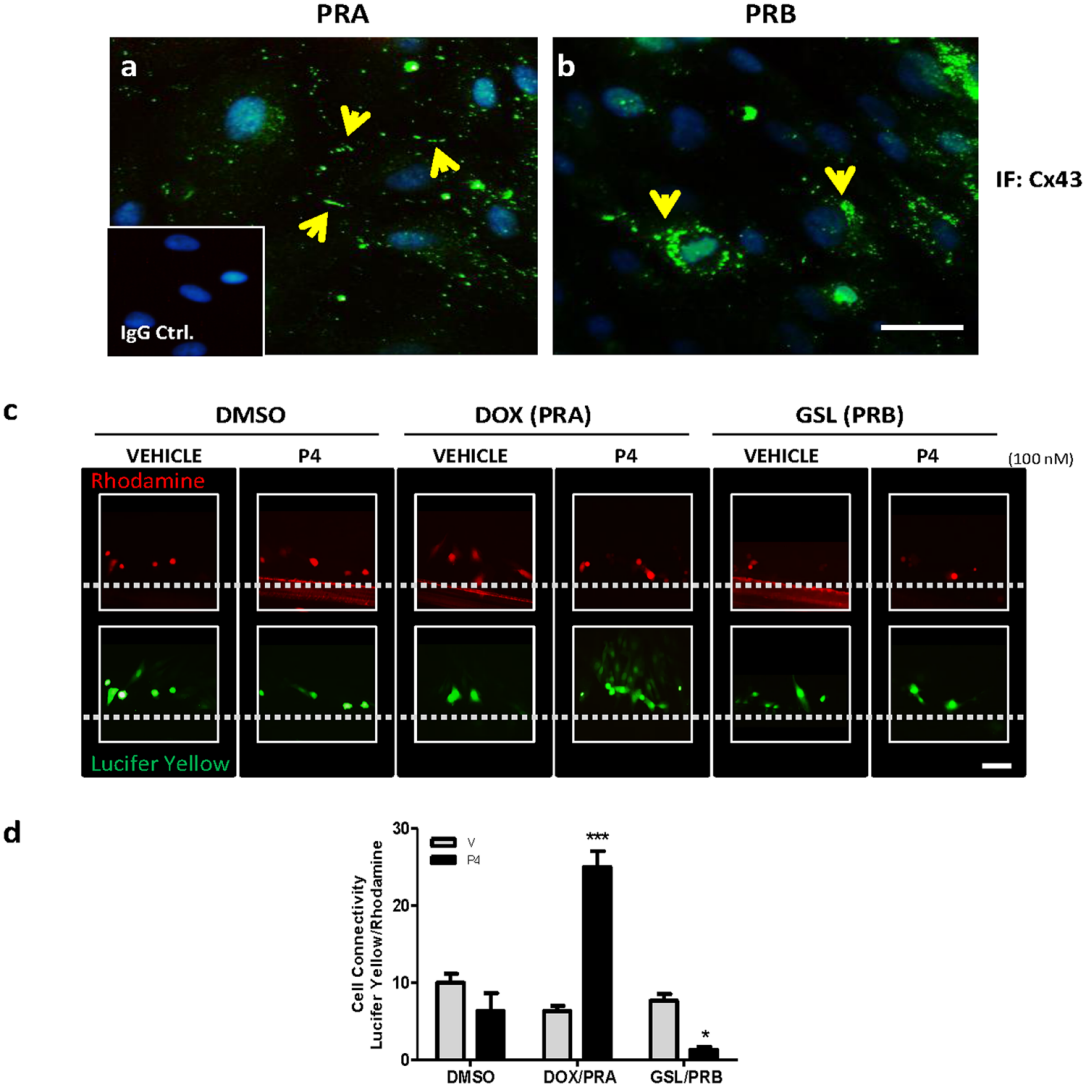
P4-Liganded PRA Promotes while P4-Liganded PRB inhibits Gap-Junction Coupling in Uterine Myocytes. (**a**,**b**) Representative Cx43 immunofluorescence images in PR inducible hTERT-HM^A/B^ cell line induced for PRA (**a**) or PRB (**b**) expression and stimulated with 100 nM P4 for 24 h. Yellow arrows show major accumulation site of Cx43 (**c**) Dye transfer assay performed with lucifer yellow (green) and rhodhamine dye (red) in hTERT-HM^A/B^ cell lines induced for PRA or PRB expression and treated with P4 (100 nM) or vehicle for 24 h. (**d**) Quantification of scrape loading/dye transfer assay (5 fields, 3 replicates, repeated four times) is shown. Data represents mean ± SD, n = 4. ‘***’ denote statistical significance at *p* < *0.001*, ‘**’ at *p* < *0.01* and ‘*’ at *p* < *0.05* with respect to vehicle control in DMSO group.

The hTERT-HM^A/B^ immortalized human myometrial cell line in which PRA and PRB expression can be controlled by independent inducers was used to selectively enhance the expression of PRA or PRB in the presence or absence of P4 and effects on GJ formation and function were examined by immunofluorescence (IF) and dye transfer analysis, respectively. We observed a striking difference in the localization of Cx43 protein. In cells expressing PRA, Cx43 was localized to the PM at the cell-cell borders **(**Fig. 1a **)** whereas in cells expressing PRB, Cx43 was restricted to the perinuclear region (Fig. 1b). Cx43 protein showed a co-localization with a tight junction associated protein Zonula occludens**-**1 (ZO-1) across the PM throughout the PRA expressing cells (Suppl. Figure 1c) but remained close to the perinuclear area in PRB cells (Suppl. Figure 1f). To examine if the PM localization of Cx43 indeed results in the formation of functional GJ channels in PRA expressing cells, we performed the scrape-loading/dye-transfer assay using lucifer yellow (MW = 457, transmits through GJ channels) and Rhodamine Dextran (MW = 10000, unable to transmit through GJ channels and stains the wounded cells only). We found that the P4 via PRA increased lucifer yellow dye transmission between neighboring cells indicating the presence of functional GJ channels (*p* < *0.0001*). In contrast, dye-transfer was significantly (*p* < *0.01*) inhibited by P4 via PRB (Fig. 1c,d).

### P4-Liganded PRB blocks forward trafficking of Cx43 from ER to Golgi

**Figure 2:**
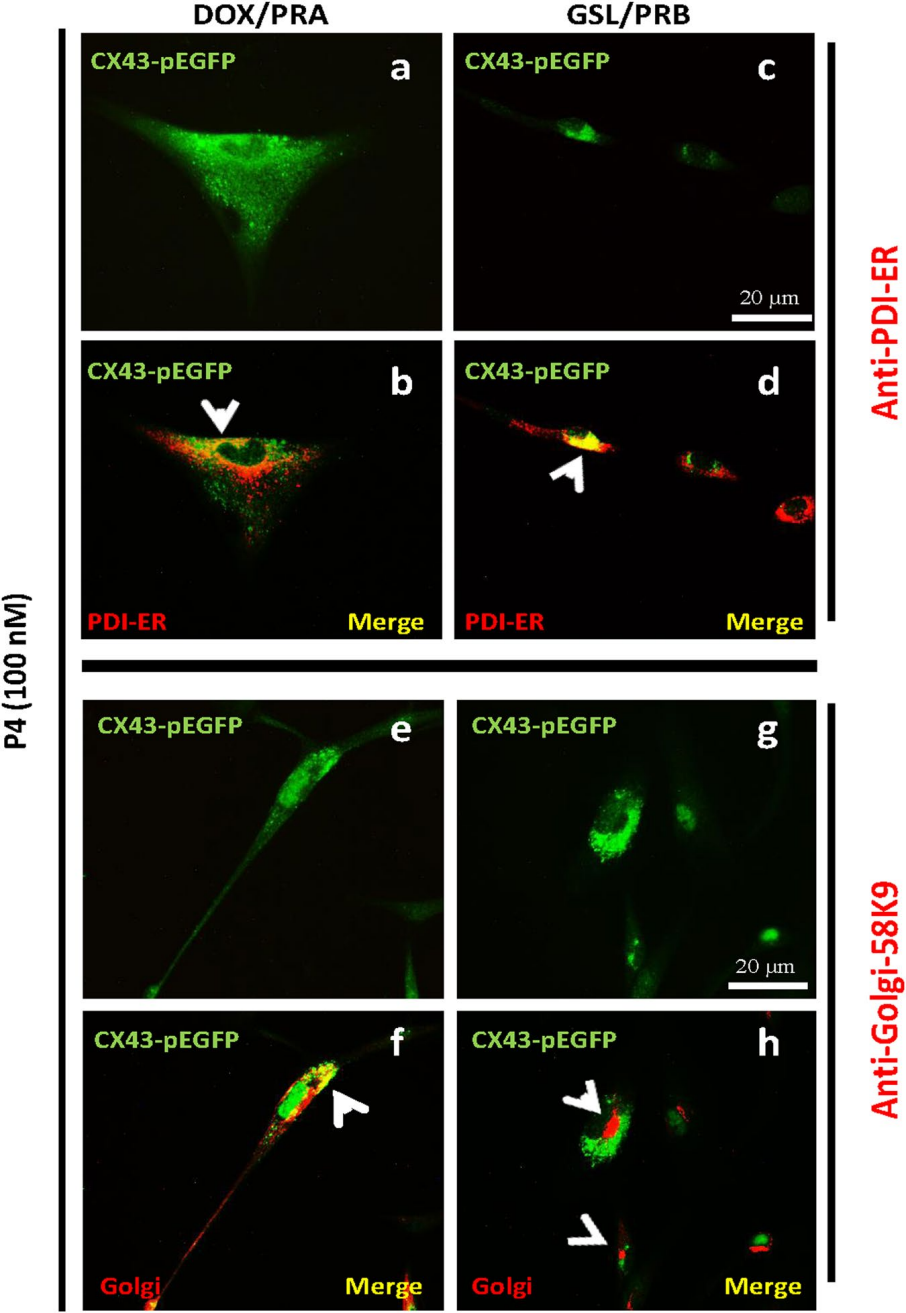
Cx43 forward trafficking from ER to Golgi is blocked in PRB expressing cells. Representative images of immunofluorescence on hTERT-HM^A/B^ cells induced for PRA (left panel) or PRB (right panel) expression and stimulated with P4 (100 nM). Images show Cx43 in green fluorescence (**a,c,e,g**), Endoplasmic reticulum (ER) marker(PDI, **b,d**) and Golgi (G) marker (Golgi-58K9, **f,h**) in red colour, and co-localization of Cx43 and ER/G in yellow [Cx43 + ER (**b,d**) and Cx43 + G (**f,h**)]. PRB expressing cells show accumulation of Cx43 in ER (**d**, yellow) but not in Golgi (**h**, red) in the merged images. Scale bar = 20 μm.

The intracellular trafficking of Cx43 protein is regulated at every step, including its transfer from ER to Golgi, from Golgi to trans-Golgi vesicles and then to the PM. Blockade of any of these trafficking steps would prevent the GJ formation, resulting in Cx43 intracellular (i.e. perinuclear) accumulation. To identify the Cx43 traffic-limiting step in hTERT-HM^**A/B**^ cells, we examined the sub-cellular localization of Cx43 by using co-immunofluorescence of Cx43 with an ER protein marker (PDI) or a Golgi marker (Golgi Protein-58K-9). We found that Cx43 was detectable in the ER of cells expressing PRA or PRB and exposed to P4 (Fig. 2b,d, yellow). However, while in P4/PRA cells, Cx43 was partially co-localized to Golgi (Fig. 2f, yellow), in P4/PRB cells Cx43 was completely excluded from the Golgi (Fig. 2h, red) indicating that ER to Golgi transport is inhibited by P4/PRB in hTERT-HM^**A/B**^ cells.

### Abundance of Cx43 isoforms is differentially modulated by P4/PRs in myometrial cells

**Figure 3:**
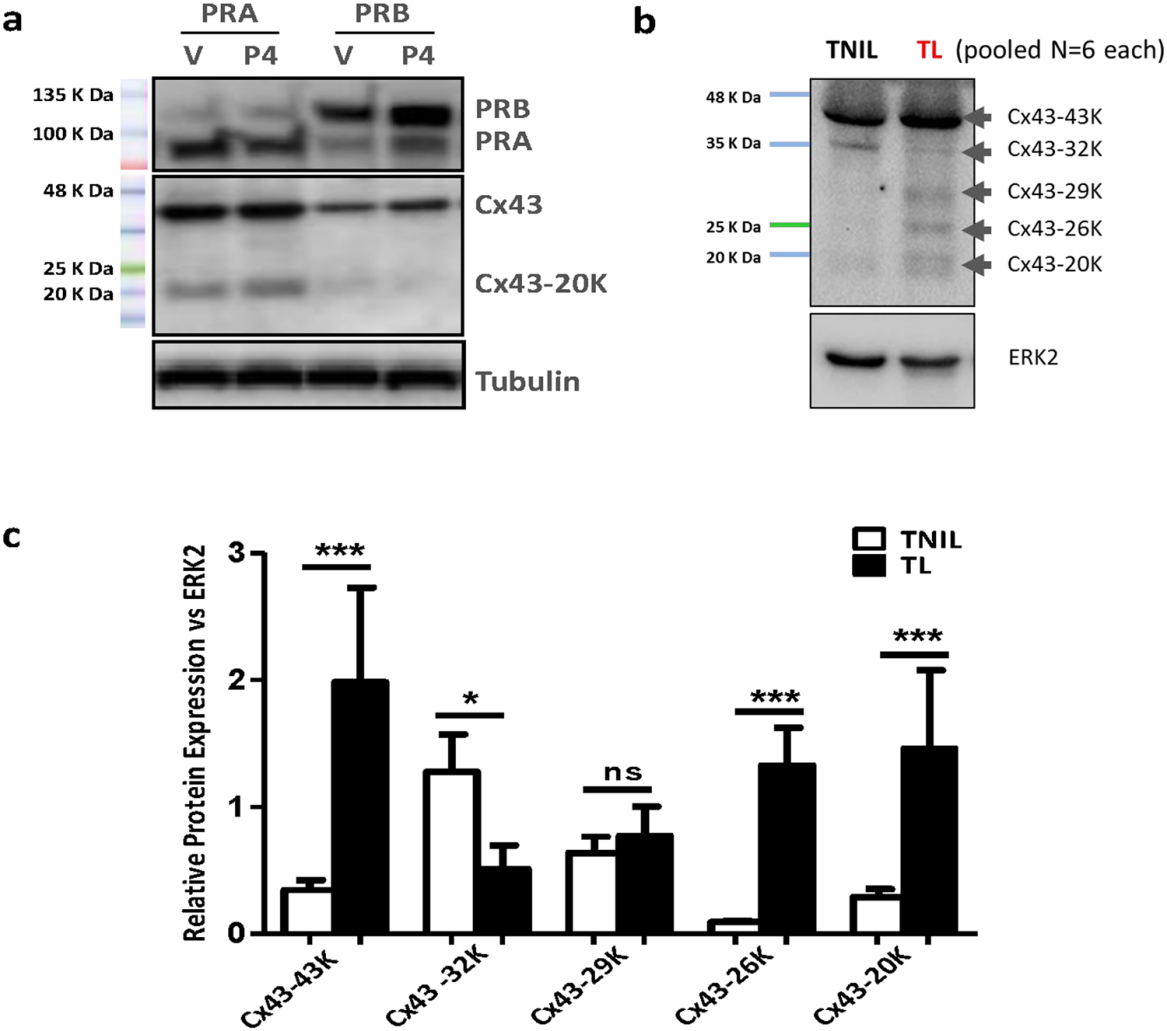
Cx43-20 K expression is upregulated in the PRA expressing cells and labouring human myometrium. Representative Western blots from (**a**) human myometrial cells hTERT-HM^A/B^ induced for PRA or PRB expression and stimulated with Vehicle or P4 (100 nM) for 24 h, and **(b)** human myometrium tissue lysates from term non-labouring (TNIL) and labouring women (TL) pooled from n = 6 samples each. Cx43 full length and Cx43-20 K (20KDa) were detected in the cell line while Cx43-43 K, 32 K, 29 K, 26 K, and Cx43-20 K were detected in the human myometrium tissues using anti-Cx43 (Millipore, Cat # AB1728). ERK2 was used as loading control. (**c**) Quantitative analysis of Cx43 isoforms in TNIL and TL. Densitometry was performed on western blots for individual TNIL and TL samples (n = 6 per group). Unpaired ttest determined the statistically significant differences in Cx43 isoforms’ expression among TNIL and TL myometrial samples. Data represents mean ± SD, n = 6/group. ‘***’ denote significant increase at *p* < *0.0001*, ‘**’ at *p* < *0.001* and ‘*’ at *p* < *0.05* in TL compared to TNIL.

Western blot analysis using an anti-Cx43 antibody that recognizes the Cx43 C-terminal domain was used to measure the abundance of full Cx43-FL and its shorter isoforms in hTERT-HMA/B cells and in human myometrium obtained from labouring and non-labouring women undergoing cesarean-section delivery at term. In hTERT-HMA/B cells only Cx43-FL and Cx43-20 K were detected. Abundance of Cx43-FL and Cx43-20 K were high in cells expressing PRA and low in cells expressing PRB **(**Fig. 3a **)**. In the term human myometrium, in addition to the Cx43-FL, four short isoforms of Cx43 including Cx43-32 K, Cx43-29 K, Cx43-26 K and Cx43-20 K, were detected. The onset of labour in human was found associated with a significant increase (*p* < *0.0001*) of Cx43-FL, 26KDa and 20KDa and a significant decrease (*p* < *0.05*) in 32KDa Cx43 isoforms in labouring myometrium compared to non-labouring (Fig. 3b,c). We also observed that the short isoforms of Cx43 (20KDa and 26KDa in particular) are hyper phosphorylated at serine residues during labour (Suppl. Figure 2).

### Exogenous expression of Cx43-20 K restores Cx43 trafficking to PM in PRB expressing cells

**Figure 4:**
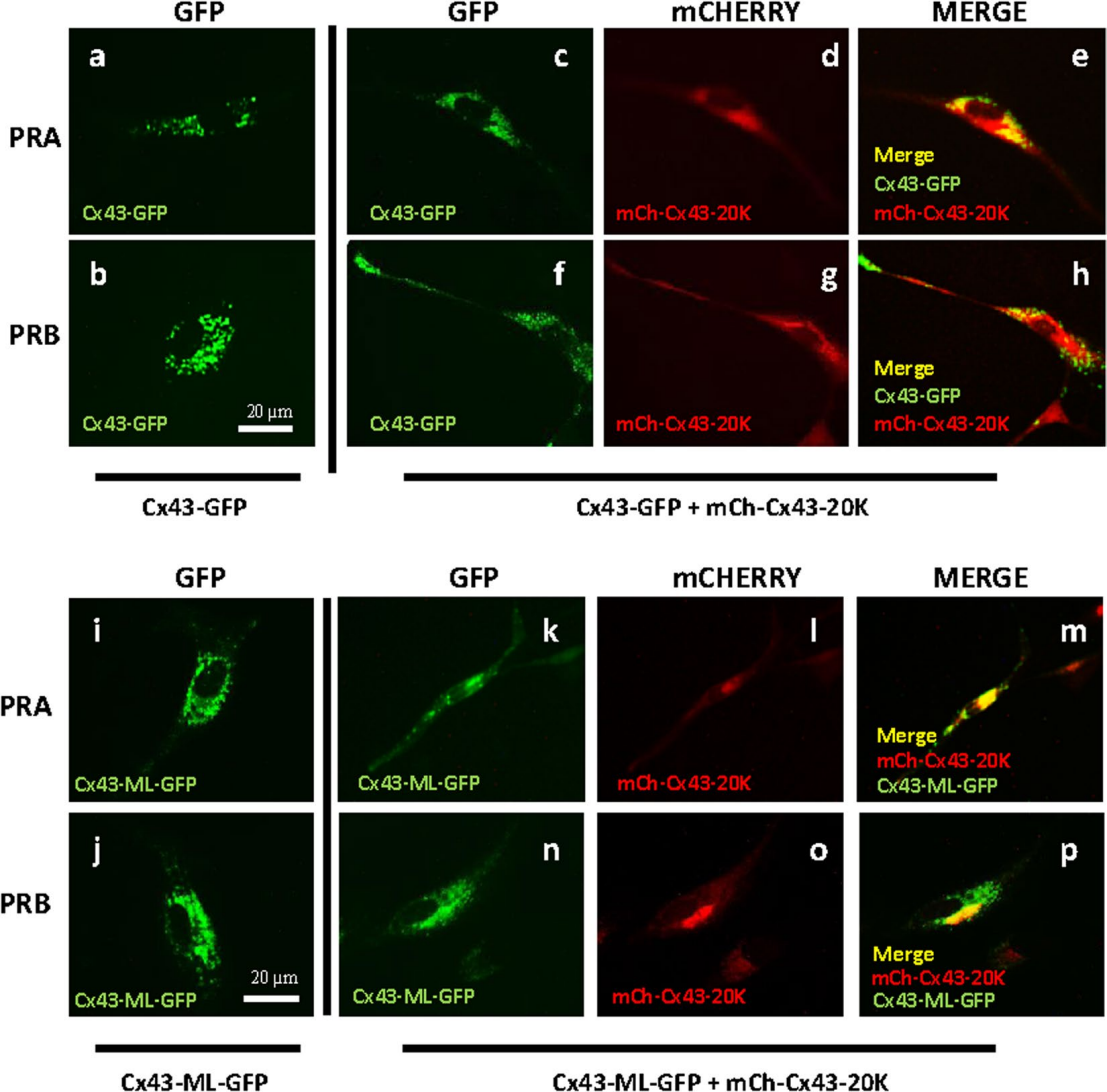
Cx43 forward trafficking is modulated by Cx43-20 K in PRA and PRB Cells. Representative images of human myometrial cells hTERT-HM^A/B^ transfected with Cx43-GFP (**a,b**) or Cx43-GFP + mCh-Cx43-20 K (**c–h**) or Cx43-ML-GFP (**i,j**) or Cx43-ML-GFP + mCh-Cx43-20 K (**k–p**) and induced for PRA (**a,c,d,e,i,k,l,m**) or PRB (**b,f,g,h,j,n,o,p**) expression for 24 h and stimulated with P4 (100 nM) for 2 h. Results showed that Cx43 forward trafficking is restricted in the PRB cells transfected with Cx43-GFP (**b**) however it was rescued by co-transfection of Cx43-GFP and mCh-Cx43-20 K (**f,g,h**). The lower panel shows that the forward trafficking of Cx43 is blocked in both PRA and PRB cells when transfected with Cx43-ML-GFP (which is unable to express Cx43-20 K, **i,j**), while it is rescued when Cx43-ML-GFP is co-transfected with mCh-Cx43-20 K (**k–p**). GFP is represented with green fluorescence, mCh-Cx43-20 K with red and their co-localization with yellow colour. Scale bar = 20 μm.

We hypothesized that disabled trafficking of Cx43-FL in P4/PRB cells is due to a lower expression of Cx43-20 K isoform and to the lack of Cx43-20 K chaperoning function. GFP-tagged-Cx43 (Cx43-GFP), when expressed in hTERT-HMA/B cells, clearly indicated that in PRA expressing cells, Cx43 was detectable throughout the cytoplasm and along the PM (Fig. 4a), while in PRB expressing cells the localization was exclusive to the perinuclear area (Fig. 4b). To increase the expression of Cx43-20 K, mCHERRY-Tagged-Cx43-20 K (mCh-Cx43-20 K or its empty vector) were co-expressed with Cx43-GFP in PRA (Fig. 4c–e) or PRB expressing cells (Fig. 4f–h), treated with P4 (100 nM) for 24 hours and the trafficking of GFP-tagged Cx43-FL was examined by GFP imaging. Over-expression of Cx43-20 K in PRB expressing cells restored the forward trafficking of Cx43-FL to the PM (Fig. 4f,h) similar to that observed in PRA expressing cells (Fig. 4a,c,e). Sub-cellular localization analysis of Cx43-20 K protein was performed by transfecting the hTERT-HM^A/B^ cells with mCh-Cx43-20 K and executing IF-based co-localization of Cx43-20 K with Golgi and ER markers (Suppl. Figure 3). Our results confirm that Cx43-20 K protein is restricted to the ER and Golgi as reported by others suggestive of its chaperoning function for Cx43-FL transport from ER to Golgi.

### Inhibition of Cx43-20 K in PRA expressing cells abrogates Cx43 forward trafficking to the PM

To further confirm that Cx43-20 K protein expression is a key limiting factor for Cx43-forward trafficking in myometrial cells, we overexpressed a GFP-tagged Cx43-FL with mutations at all internal translation initiation sites (Cx43-ML-GFP) such that the shorter Cx43 isoforms are not produced. We found that deficiency of Cx43-20 K inhibited trafficking of Cx43-FL regardless of PR isoform expression (Fig. 4I,j). This effect was rescued by the co-expression of mCh-Cx43-20 K with Cx43-ML-GFP (Fig. 4k–p).

### P4-Liganded PRs differentially regulate Cx43 forward trafficking in HEK-293T cells

To explore the specificity of this phenomenon to uterine muscle, experiments were replicated in human embryonic kidney cells (HEK-293T, ATCC® CRL3216™) which do not express endogenous PRs. We over-expressed PRA or PRB in HEK-293T cells along with GFP-tagged Cx43 and examined the trafficking of Cx43 by imaging GFP. Similar to the myometrium we found that in response to P4, PRA promotes while PRB inhibits Cx43 forward trafficking in HEK-293T cells (Suppl. Figure 4a). Moreover in HEK-293T cells expressing PRA, transport of the Cx43-ML-GFP mutant to the PM was inhibited due to a lack of Cx43-20 K suggesting that Cx43-20 K is central to the PRA-mediated Cx43 forward trafficking (Suppl. Figure 4b). Similar to the hTERT-HM^A/B^ cells, restoration of Cx43-20 K expression by the co-expression of mCh-Cx43-20 K repaired the Cx43 trafficking defect in PRB expressing cells (Suppl. Figure 5b), confirming that PR isoforms differentially regulate Cx43 forward trafficking/GJ formation irrespective of cell type and that Cx43-20 K chaperoning function is not specific to uterine myocytes alone.

### P4/PRA regulates Cx43-20 K expression through Cap-Independent translation via inhibition of the mTOR signaling Pathway

**Figure 5:**
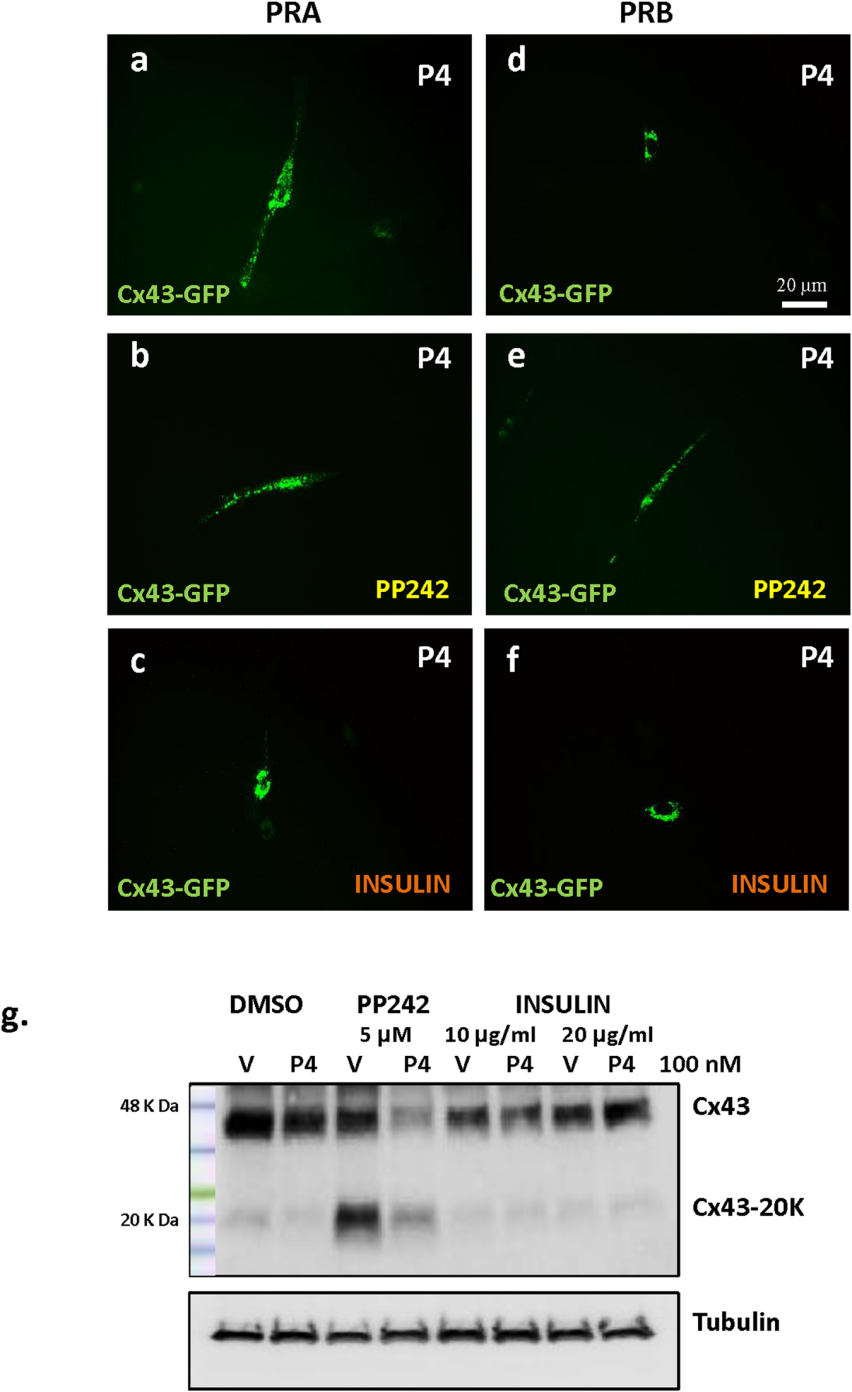
Cx43 forward trafficking and Cx43-20 K expression is regulated by mTOR signaling pathway in human myometrial cells. Representative images of hTERT-HM^A/B^ cells transfected with Cx43-GFP and induced for PRA (**a,b,c)**, or PRB (**d,e,f**) expression for 24 h and stimulated with P4 (100 nM) for 2 h. Results showed that Cx43 forward trafficking is promoted in PRA cells (**a**) and restricted in the PRB cells (**d**) however inhibition of mTOR signaling pathway via specific inhibitor PP242 (5 μM) resulted in the rescue of Cx43 trafficking defect in PRB cells (**e**) whereas stimulation of mTOR signaling pathway via insulin (10 μg/ml) treatment resulted in the inhibition of Cx43 trafficking in PRA cells (**c**). Scale bar = 20 μm. Regulation of Cx43-20 K expression in PRB cells by PP242 and insulin is shown in the western blot image (**g**).

Since it was reported that the process of cap-independent translation (which generates N-terminally truncated short isoforms of Cx43), is negatively regulated by the mTOR signaling pathway, we manipulated this pathway using a mTOR specific inhibitor (PP242) or stimulator (insulin) in hTERT-HM^**A/B**^ cells induced to express PRA (Fig. 5a–c) or PRB (Fig. 5d–f) and examined the changes in forward trafficking of Cx43-FL to the PM. The inhibition of mTOR signaling by PP242 (5 μM) did not influence Cx43 protein distribution in cells expressing PRA (Fig. 5b compared to 5a), but led to the restoration of Cx43 trafficking to the PM in cells expressing PRB (Fig. 5e compared to 5d). Importantly, the stimulation of mTOR by insulin treatment (10 μg/ml) resulted in the inhibition of Cx43 forward trafficking even in PRA expressing cells (Fig. 5c compared to 5a), while it did not change the perinuclear localization of Cx43 in PRB expressing cells (Fig. 5f compared to 5d). Western blot analysis showed that the Cx43-20 K expression in hTERT-HMA/B cells was induced by PP242 and inhibited by insulin (Fig. 5g and Suppl. Figure 6a) suggesting that the PRs regulate Cx43-20 K expression by influencing the internal translation of Cx43 transcript such that PRA facilitates cap-independent translation via inhibition of mTOR signaling pathway while PRB inhibits cap-independent Cx43 translation.

Recently it was reported that intra-membrane proteolytic cleavage by gamma secretase can also contribute to the Cx43-20 K expression^35^. We examined the expression of Cx43-20 K in hTERT-HMA/B cells treated with gamma secretase inhibitor (ZLL2, 50 μM), however a decrease in Cx43-20 K expression was not observed (Suppl. Figure 6b), suggesting that proteolytic cleavage does not account for the increased expression of Cx43-20 K isoform in human myometrial cells expressing PRA.

### PRA/B do not regulate 14-3-3θ expression or its interaction with Cx43

To explore the putative molecular mechanism linking Cx43 trafficking with PRA/PRB we examined 14-3-3θ protein expression in hTERT-HM^**A/B**^ cells treated with P4 (100 nM), There was no difference detected in PRA vs PRB expressing cells. Next, Proximity Ligation Assay (PLA) analysis was performed to determine the interaction between Cx43-FL and 14-3-3θ proteins, however no significant difference between PRA versus PRB cells was determined in the interaction between Cx43-FL and 14-3-3θ (Suppl. Figure 7b).

## Discussion

Uterine myocyte connectivity is critical to ensure their ability to generate the synchronous myometrial contractions needed to expel the fetus during labour. It has been established that in parallel with the synthesis of Cx43, it’s trafficking to and incorporation into the PM is a critical event for labour initiation. Our study challenges the current dogma that P4 inhibits Cx43 synthesis and GJ formation and provides compelling evidence that P4 presents contrasting actions via PRA and PRB such that via PRB it inhibits while via PRA it promotes Cx43 trafficking and GJ formation in human myometrium. Both PR isoforms are equally expressed in the human myometyrium, thus their contrasting response to P4 may depend on the expression ratio of PRA/PRB. Our study proves that both PRA and PRB receptors intricately mediate the actions of P4 in human myometrium during pregnancy and labour such that PRB maintains the myometrial quiescence during pregnancy while PRA is capable of transforming the myometrium from dormant state to an actively contracting organ by enhancing the myocyte connectivity during labour. We previously reported that the expression of Cx43 in myocytes during pregnancy and labour is directly regulated by P4 such that during pregnancy in the P4-liganded state PRB dominates the genomic function and suppresses Cx43 gene transcription, while at term, the unliganded PRA dominates the genomic function and activates Cx43 transcription^34^. We also found that PRs have different cellular localization depending on their unliganded or P4-liganded state such that in its unliganded state PRA is nuclear while PRB is cytoplasmic and in P4-liganded state PRB is nuclear while PRA is cytoplasmic. This study was conducted following the observation of striking differences in the localization of Cx43 in PRA versus PRB expressing myocytes in the presence of P4. In PRA expressing cells Cx43 was detected in the cytoplasm and at the PM between the myocytes while in PRB cells it was peculiarly restricted to the perinuclear region. We further determined that the P4-liganded PRA promotes forward trafficking of Cx43-FL towards PM and formation of functional GJs while P4-liganded PRB inhibits Cx43 trafficking even if Cx43 is exogenously over expressed. We concluded therefore that GJ formation is regulated positively by PRA and negatively by PRB. Previously it has been reported that Cx43 is expressed immediately prior to labour (whether term or preterm), followed by the rapid formation of GJs between adjacent myocytes and their abrupt removal post-partum^20,26^. Our current data show that the increased expression of proteins, Cx43-FL and its short isoforms Cx43-20 K, in labouring myometrium is coincident with increased expression of PRA^30^. The Cx43-20 K transcript generated from the internal translation process is a trafficking chaperon for the Cx43-FL^19^. Here we report that in response to P4, PRs differentially regulate the expression of Cx43-20 K isoform in myometrial cells such that PRA promotes while PRB inhibits its expression. Taken together our data suggest that unliganded nuclear PRA, which we previously showed increases in myometrial cells with the onset of labour, promotes Cx43 transcription to ensure the availability of protein while P4-liganded cytoplasmic PRA increases forward trafficking of Cx43 to the PM, ensuring the GJ formation and myocytes connectivity – an ultimate requirement for labour onset.

The detailed molecular mechanism linking Cx43 trafficking in myometrial cells to PRA/PRB is still unknown. Amongst the reported regulators of Cx43 forward trafficking is a protein 14-3-3θ^36^. Cx43 is the only connexin with a 14-3-3θ binding motif ^37^ and its interaction with 14-3-3θ protein masks the Arg-based ER-retention signal on the Cx43 molecule, hence facilitating the forward trafficking of Cx43 from ER to the trans-Golgi Network (TGN) in Rat-1 fibroblast cells^36,38^. We examined this potential regulatory mechanism in myometrial cells in relation to PRA/PRB overexpression, however no effect of PRA or PRB on 14-3-3θ expression or its interaction with Cx43 was detected (Suppl. Figure 7b).

We explored two potential mechanisms regulating the expression of Cx43-20 K isoform in myometrial cells; 1) mTOR signaling pathway, which is an inhibitor of cap-dependent translation and hence Cx43-20 K expression and 2) gamma secretase-mediated intra-membrane proteolytic cleavage of Cx43, which generates Cx43-20 K isoform. We determined that the Cx43-20 K expression is not regulated via intra-membrane proteolytic cleavage in PRA expressing cells however the stimulation of mTOR signaling suppressed the Cx43-20 K expression in PRA while mTOR inhibition enhanced Cx43-20 K expression in PRB cells. Interestingly, we have previously reported a decline in the activity of mTOR signaling in rat myometrium during late gestation and term labour^39^, which is coincident with the increased expression of Cx43-20 K during labour (as shown in Fig. 3b,c).

**Figure 6:**
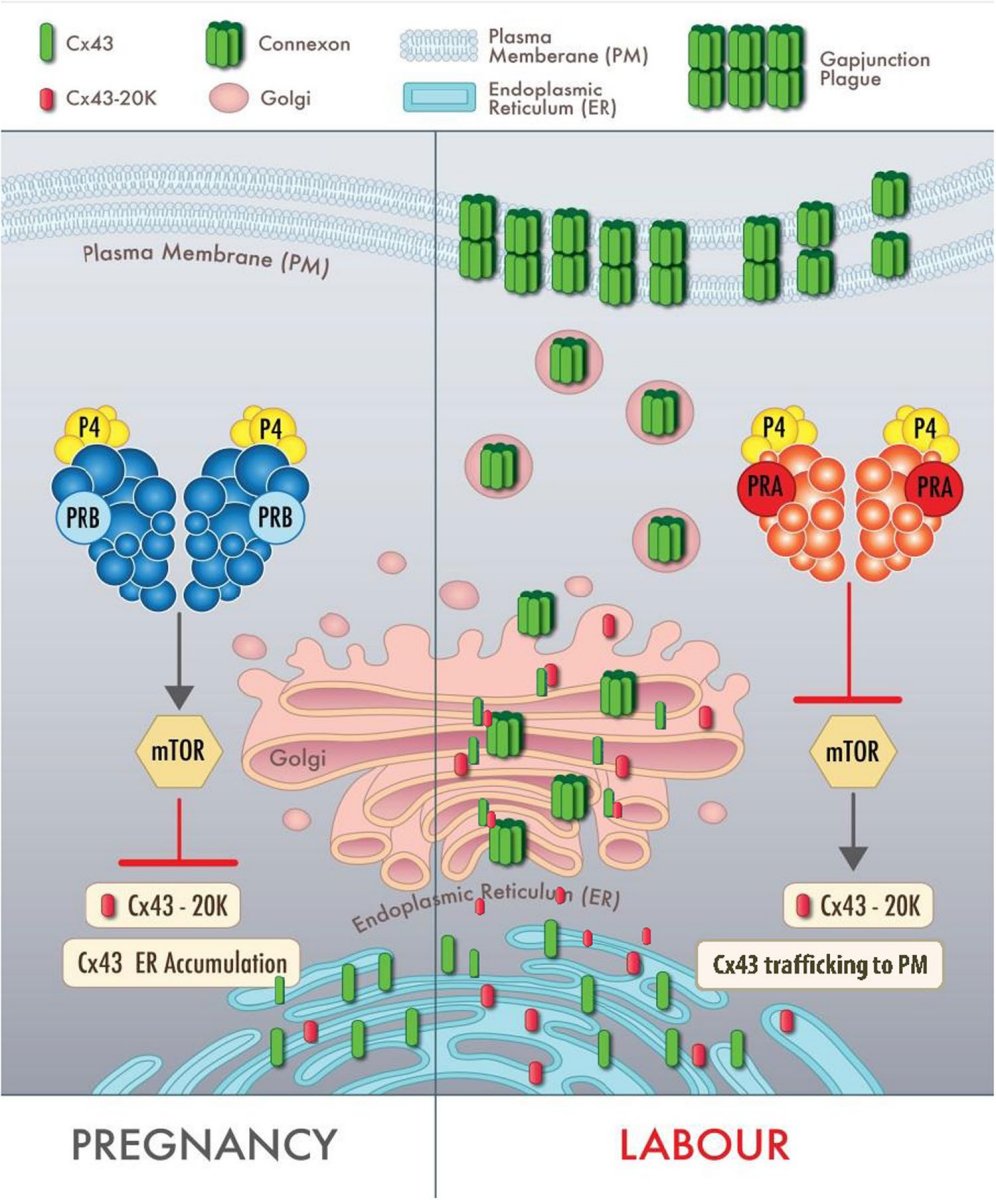
Hypothetical model of regulation of Cx43 forward trafficking and GJ formation by PR isoforms in human myocyte during pregnancy and labour. Model shows that during pregnancy P4-liganded PRB inhibits forward trafficking of Cx43/GJ formation by inhibiting Cx43-20 K expression/translation which results in accumulation of Cx43 in the ER and no cell-cell connectivity between myocytes. During labour P4-liganded PRA promotes Cx43 forward trafficking/GJ formation by inducing Cx43-20 K expression (which facilitates ER to Golgi transport of Cx43) and ensuring cell-cell connectivity to synchronize myometrial contractions during labour.

It is well-established that labour is associated with increased myometrial cell-to-cell connectivity through GJ formation. The regulation of Cx43 expression and GJ formation in myometrium of rodents has been attributed to precipitous fall of peripheral P4 level as a result of luteolysis in a high E2 background. However, in women the changes in Cx43 expression and GJ formation occur at the time of labour without a fall in maternal P4 levels. Our earlier data showed that during pregnancy P4-liganded PRB suppresses Cx43 transcription, whereas in the transition towards labour, increased nuclear metabolism of P4 leads to domination of unliganded PRA in the myometrial nuclei and activation Cx43 transcription^34^. Here we report that not only does PRB suppress Cx43 transcription but it also inhibits Cx43 forward trafficking and GJ formation. On the other hand PRA not only increases the transcription of Cx43 gene but it also enhances the forward trafficking of Cx43 protein by facilitating the cap-independent translation of Cx43-20 K isoform, to ensure GJ formation and myocyte coupling. We propose a new model of the complex molecular regulation of intracellular Cx43 transport during pregnancy and labour in human myometrial cells (Fig. 6). We speculate that during pregnancy Cx43 gene expression is largely silenced and any Cx43 that is synthesized accumulates in the ER of uterine myocytes. P4-liganded PRB inhibits forward trafficking of Cx43 and formation of functional GJs by preventing the expression/translation of the short isoform-chaperon protein: Cx43-20 K, which results in low cell-cell connectivity between myocytes and myometrial quiescence during pregnancy. During labour unliganded nuclear PRA increases Cx43 transcription, while P4-liganded cytoplasmic PRA induces Cx43-20 K expression, which facilitates ER to Golgi transport of Cx43, promotes Cx43 forward trafficking to PM, GJ formation and ensures cell-cell connectivity to synchronize myometrial contractions and labour induction (Fig. 6). The fact that the action of PRA is P4-dependent suggests that this regulation is mediated by P4-liganded cytoplasmic PRA through non-genomic mechanisms, possibly by cross-talk with other signaling pathways (e.g., mTOR). Taken together our study indicates that in myometrial cells PRA is a master regulator of Cx43 expression, it’s trafficking, and formation of functional GJ channels which is essential for myocyte coupling and induction of labour.

## Materials and Methods

### Cell lines and cell culture

Human myometrial cell line with telomerase immortalization, DOX inducible PRA and RheoSwitch ligand (RSL)-inducible PRB expression system (hTERT-HM^A/B^), as previously described^40^, was used in this study. Medium for this cell line DMEM/Ham’s F12 (1:1) containing 2 mM L-Glutamine was further supplemented with 5% charcoal stripped fetal bovine serum (FBS), 0.5% penicillin/streptomycin, 100 µg/ml of Geneticin, 1 µg/ml of Hygromycin B and 5 µg/ml Blasticidin. All the reagents for cell culture were purchased from Invitrogen Canada Inc. Cells were maintained at 37 °C with 5% CO_2_. We used 25 ng/ml DOX and 100 nM GSL to induce PRA or PRB expression respectively.

### Scrape loading/Dye-transfer Assay

We followed the previously published protocol^41^ with slight modifications, for scrape loading/dye transfer assay to determine the GJ functionality. Briefly, the hTERT-HM^A/B^ cells plated in 6-well culture plate were induced for PRA or PRB receptor expression and grown to form a monolayer (~90% confluence). Cells were starved for 4–6 hours and then stimulated with P4 (100 nM) or its vehicle (70% ethanol) and DOX (for inducing PRA expression) or GSL (for inducing PRB expression), for 24 hrs. Cells were then washed with PBS and a 1 ml mixture of 0.05% Lucifer Yellow CH (MW 457.2, able to cross GJ channels) and 0.05% Rhodamine Dextran (MW 10,000, unable to cross GJ channels and only stain wounded cells) in 1:1 ratio was added to the cell layer and a wound was scratched across the plate using a scalpel. After 2 minutes incubation with the dye mixture the excessive dye was removed and cells were washed thrice with PBS and incubated with the complete medium for additional 5 minutes in the incubator at 37 °C. Cells were then fixed with 4% PFA for 10 minutes washed with PBS and imaged around the wound with Leica DM IL LED-Inverted fluorescence microscope with micropublisher 5.0 RTV Q imaging system under 20X objective. At least 10 random fields per well were taken into analysis and the cell connectivity was analyzed from the ratio of Lucifer Yellow/Rhodamine positive cells.

### Transient transfection

Transient transfection was performed using TransIT-LT1 transfection reagent (Mirus Bio, USA) following manufacturer’s protocol. The plasmid constructs pDEST/hCx43-EGFP-N1 (Addgene plasmid #40907), pDEST-mCherry-GJA1-20K-N1 (Addgene plasmid #49861), pDEST-EGFP-Cx43ML-N1 (Addgene plasmid #49860), and their empty vector controls were kind gift from Dr Robin Shaw and their cloning procedure is described previously^19^.

### Immunofluorescence and Co-immunofluorescence

We used the immunofluorescence protocol as described before^42^. In brief cells were rinsed with ice cold PBS, fixed with ice cold Methanol/Acetone (1:1), washed thrice with PBS and permeabilized with 0.2% Triton X-100 for 5 min at room temperature. Then the cells were washed thrice again with PBS and blocked with 0.1% BSA in PBS for an hour. Incubation with primary antibodies or control IgG (1:100 in blocking solution) was performed for overnight at 4 °C. Next day, cells were washed three times with PBS and incubated with fluorochrome-labeled secondary antibodies, Alexa Fluor 594 or 488 (Molecular Probes, Thermo Fisher Scientific, USA) with 1:300 dilution in blocking solution for 30 min. Finally, cells were washed four times with PBS (10 min each) and counterstained with 1 µg/ml of DAPI. Fluorescent microscopy was performed using Leica DM IL LED-Inverted fluorescence microscope with micropublisher 5.0 RTV Q imaging system or Leica Spinning Disc Confocal Microscope under various magnifications. Primary and secondary antibodies used in this study are listed in Table 1.

**Table 1:** Sources and dilutions of antibodies used in this study.

**ANTIGEN**	**ANTIBODY**	**DILUTION**	**SOURCE**
Cx43	AB1728	1: 1000 in 1% BSA	Millipore
PR	sc-7208	1: 1000 in 5% Milk	Santa Cruz
PDI	ab31811	1: 1000 in 1% BSA	Abcam
Golgi-58K9	ab27043	1: 500 in 1% BSA	Abcam
cis-Golgi-GM-130	ab52649	1: 500 in 1% BSA	Abcam
14-3-3-θ	sc-59414	1: 500 in 5% Milk	Santa Cruz
Tubulin	T5168	1: 5000 in 5% Milk	Sigma Aldrich
ERK2	sc-154	1: 1000 in 5% Milk	Santa Cruz
ZO-1	sc-10804	1: 500 in 1% BSA	Santa Cruz
Phosphoserine	ab9332	1: 200 in 1% BSA	Abcam

### Protein extraction and immunoblotting

Cell lysates were prepared in lysis buffer (0.08 M Tris/HCl -pH 6.8, 2% SDS, 10% Glycerol) with freshly added protease and phosphatase inhibitor cocktail (Thermo Fisher Scientific Inc.) and total protein was extracted by vortexing at maximum speed, incubation on ice for 10 minutes, sonication on ice for 10″, denaturing at 95 °C for 5 min and spinning in cold centrifuge at 14,000 rpm for 25 min. Cytoplasmic and nuclear lysates were prepared with NE-PER, Nuclear and Cytoplasmic Extraction kit (Thermo Fisher Scientific, USA) as per instructions. Equal amount of protein was separated by SDS-PAGE and transferred to a polyvinylidene difluoride (PVDF) membrane (Trans-blot Turbo Midi PVDF, BioRad) using Turbo Trans-Blot system (BioRad). After blocking for an hour with 5% milk in TBS-T, the membranes were incubated with primary antibody at 4 °C for overnight. The membranes were subsequently probed with horseradish peroxidase-conjugated secondary antibody at room temperature for an hour. Signals were detected using Luminata HRP-substrate (Millipore) and imaging was performed with ChemiDoc imaging system (BioRad). Primary and secondary antibodies used in this study are listed in Table 1.

### *In-situ* Proximity Ligation Assays (PLA)

Protein-protein interactions were detected using the Duolink II *in-situ* PLA Detection Kit (Sigma Aldrich, USA). Briefly, the hTERT-HM^A/B^ were cultured on 16 well chamber slides (Lab Tek, Fisher, CA), induced for PRA or PRB expression, serum starved for 24 h in serum free medium and then stimulated with Progesterone (P4, 100 nM) for 2 h. Cells were then washed with cold PBS and fixed with cold methanol: acetone (1:1) for 3 min. After washing, cells were permeabilized with 0.2% Triton X-100 for 5 min, blocked with the blocking solution (provided in the Duolink kit) for an hour and incubated with primary antibodies for overnight at 4 °C. Hybridization with PLA probes (plus and minus), ligation and amplification of conjugants was performed as per manufacturer’s instructions. Slides were mounted with Dapi containing antifade mounting medium (Sigma Aldrich, USA) and pictures were taken at constant exposure and 200X magnification by Leica DM IL LED-Inverted fluorescence microscope with micropublisher 5.0 RTV Q imaging system.

### Statistical analysis

Differences among several groups and variables were determined by two-way analysis of variance (ANOVA), followed by Bonferoni post-test for multiple comparison using *Prism* software (GraphPad Prism; GraphPad Software, San Diego, CA).

## Electronic supplementary material

Supplementary Figures
