# Supplementary material for: Progesterone Via its Type-A Receptor Promotes Myometrial Gap Junction Coupling
Source: Sci Rep. 2017 Oct 17;7:13357. doi: 10.1038/s41598-017-13488-9 (PMC5645358; doi:10.1038/s41598-017-13488-9)
Supplement: Supplementary file 1 — Supplementary Figures [file 41598_2017_13488_MOESM1_ESM.pdf]

Progesterone Via its Type-A Receptor Promotes Myometrial Gap Junction Coupling

Lubna Nadeem, Oksana Shynlova, Sam Mesiano, and Stephen Lye

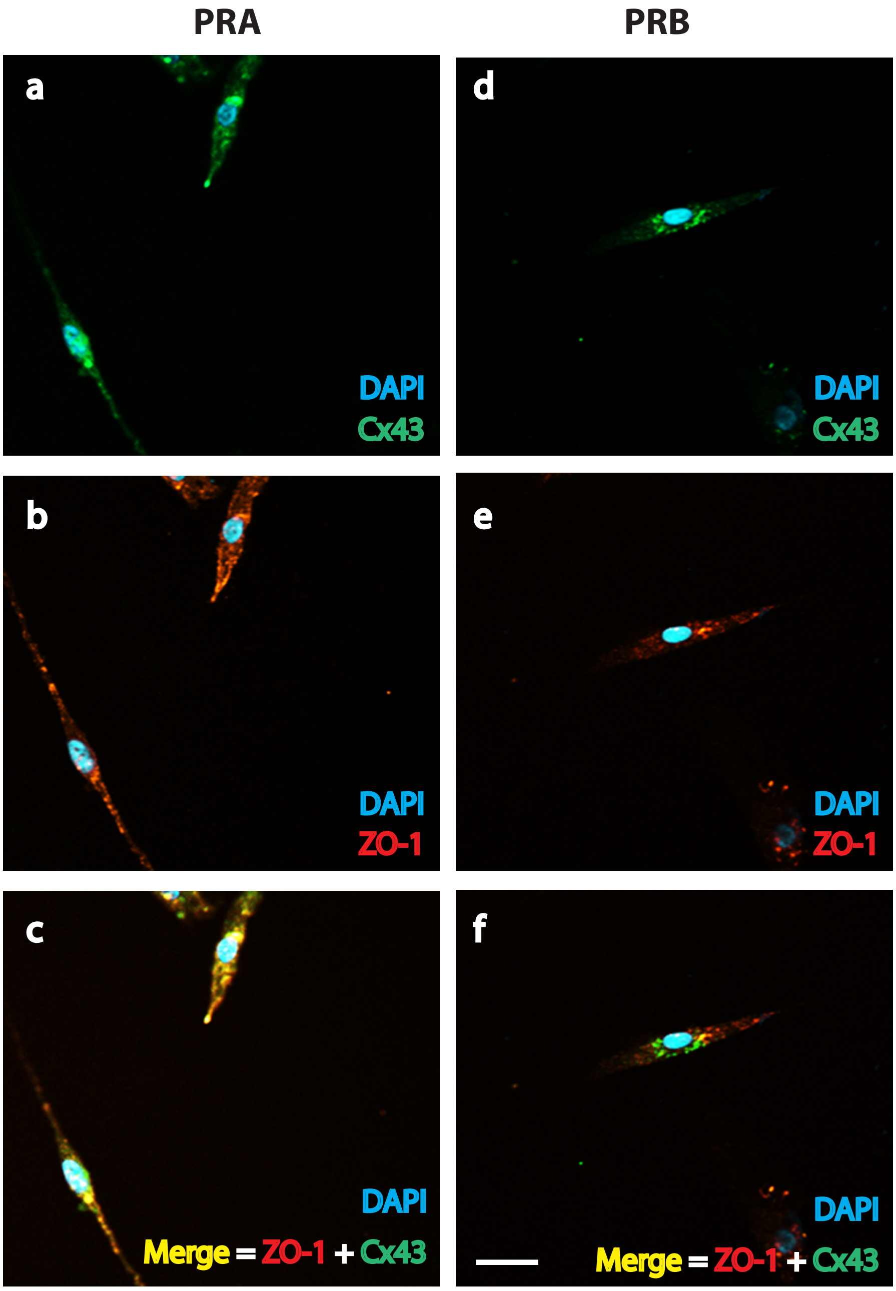

Supplementary Figure 1: PR inducible human myometrial cells were induced with DOX to express PRA **(a-c)** or with GSL to express PRB **(d-f)** and stimulated with P4 (100 nM) or its vehicle for 24 h. Immunofluorescence was performed with Anti-Cx43 and Anti-ZO-1 antibodies. Representative images are shown with Cx43 (green, **a** and **d**), ZO-1 (red, **b** and **e**) and their co-localization (yellow, **c** and **f**). Nuclei are stained with DAPI (blue). Scale bar = 20 µm.

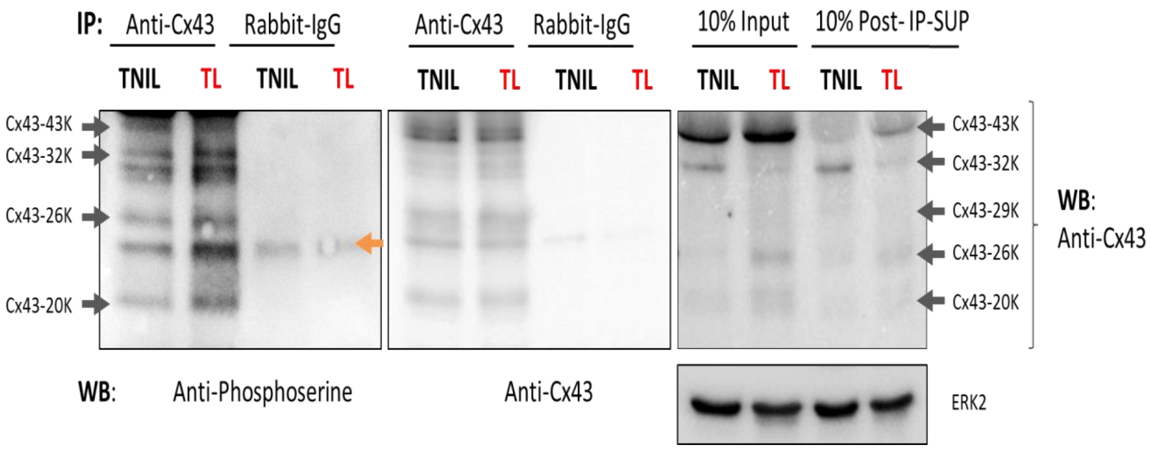

Supplementary Figure 2: Cx43 isoforms are hyper-phosphorylated during labour. a) Anti-Cx43 conjugated magnetic beads (100 ul) were saturated with Cx43 from 1 mg of protein pooled from 6 individual human myometrium tissue samples from term non-labouring (TNIL, n=6) and labouring (TL, n=6) for 2 hours at room temperature, washed thrice with PBS-T, eluted with reducing Laemmli buffer (2x) at 70^{0}C for 10 min and subjected to SDS-PAGE and immunoblotted with anti-phosphoserine. 250 µg of protein from samples was subjected to Rabbit-IgG conjugated beads as control. Result showed higher extent of Cx43 phosphorylation in TL compared to TNIL **b)** Blot '**a**' was stripped and re-probed with anti-Cx43 which shows that equal amount of Cx43 was precipitated in both TNIL and TL samples. **c)** 10 % input protein and post IP supernatant (Post-IP-SUP) from each sample was subjected to western blot under similar conditions. Post-IP-SUP shows a higher content of Cx43 isoforms in TL sample (relative ratio similar to the input) confirming that the beads were equally saturated with Cx43 from both TNIL and TL samples. **d)** Blot ''**c**" was stripped and re-probed with Anti-ERK2 antibody as loading control. Grey arrows point to various Cx43 isoforms and orange arrow points to the IgG band.

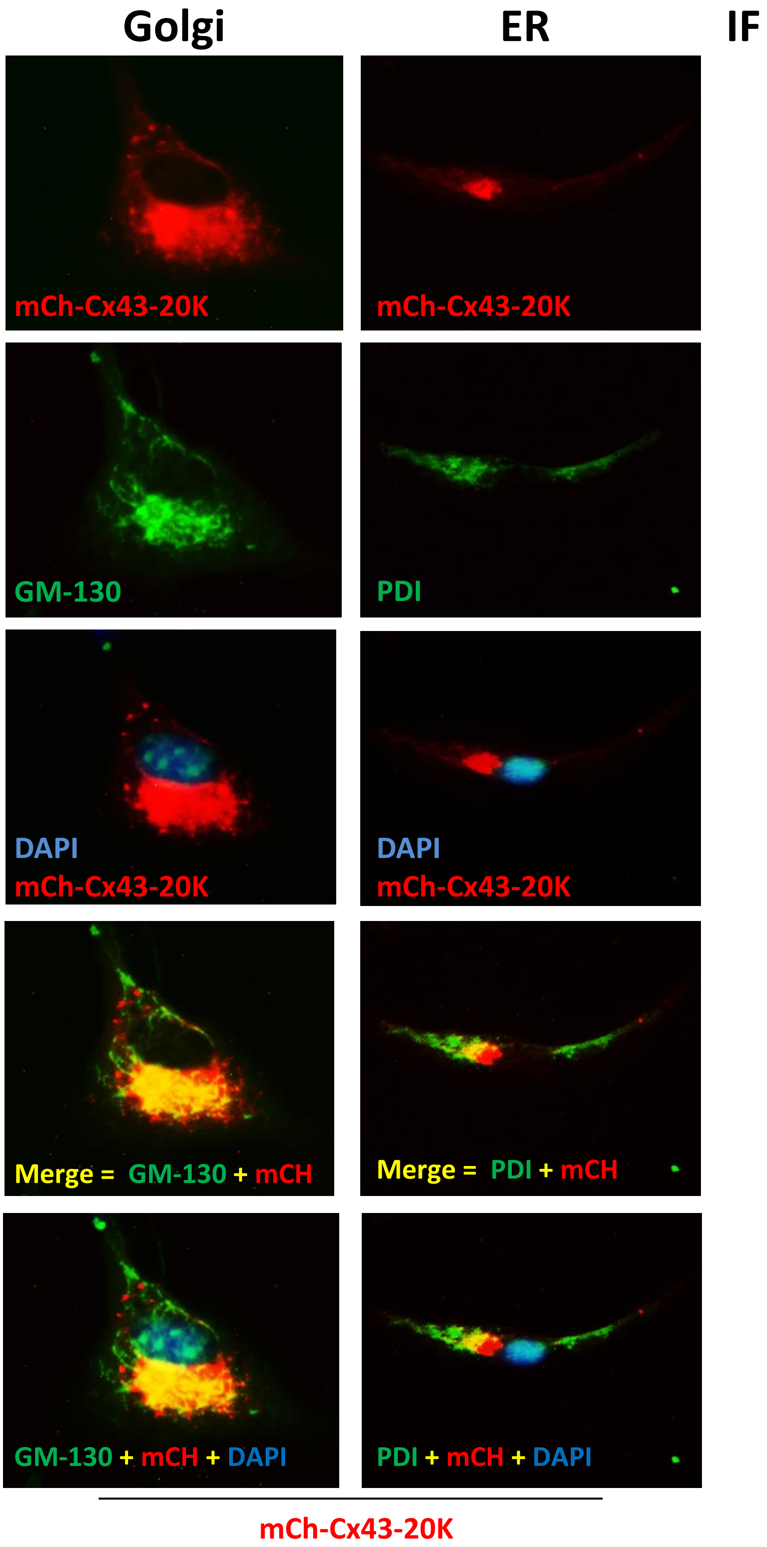

Supplementary Figure 3: hTERT-HM$^{A/B}$ cells were transfected with mCHERRY tagged Cx43-20K. Immunofluorescence was performed with cis-Golgi antibody GM-130 (left panel) or ER-marker PDI (right panel), and co-localization with mCHERRY fluorescence was performed after counter staining with DAPI. Images were taken at 200X magnification. Representative pictures of images taken with individual filters and merged pictures are shown. DAPI represents the nucleus, red fluorescence represents Cx43-20K and green fluorescence shows localization of trans golgi network respectively.

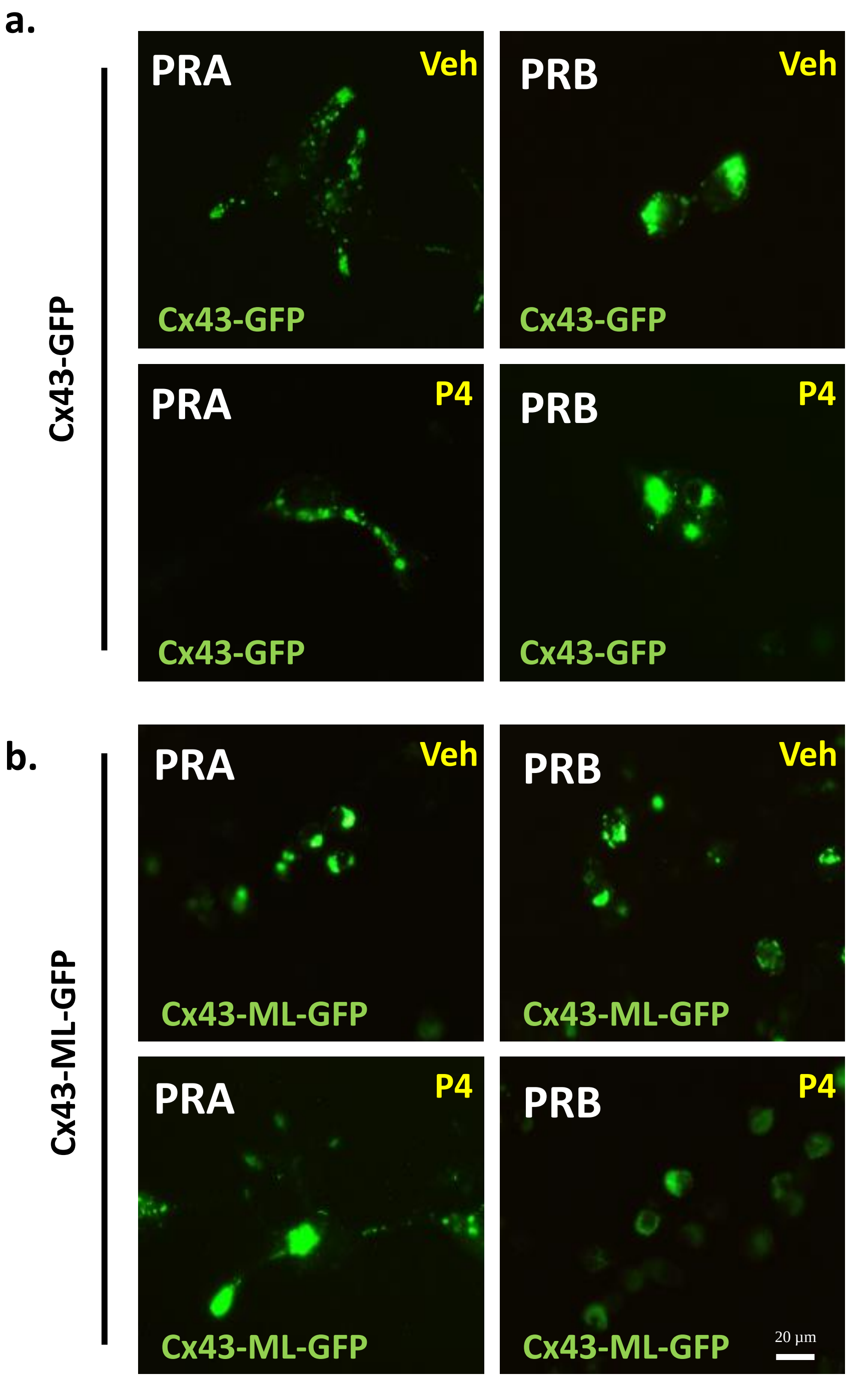

Supplementary Figure 4: PRA promotes while PRB inhibits Cx43 forward trafficking in HEK-293T cells. HEK-293T cells were transfected with PRA or PRB and Cx43-GFP **(a)** or Cx43-ML-GFP **(b)** plasmid constructs and treated with vehicle or P4 (100 nM) for 24 h. GFP images show localization of full length Cx43 (Cx43-FL). Results show that (a) PRA promotes Cx43 trafficking while PRB inhibits it and the effect was more pronounced post P4 stimulation, **(b)** when Cx43 mutant vector (unable to express Cx43 short isoforms) was expressed, PRA was unable to traffic Cx43-FL. Scale bar = 20 µm.

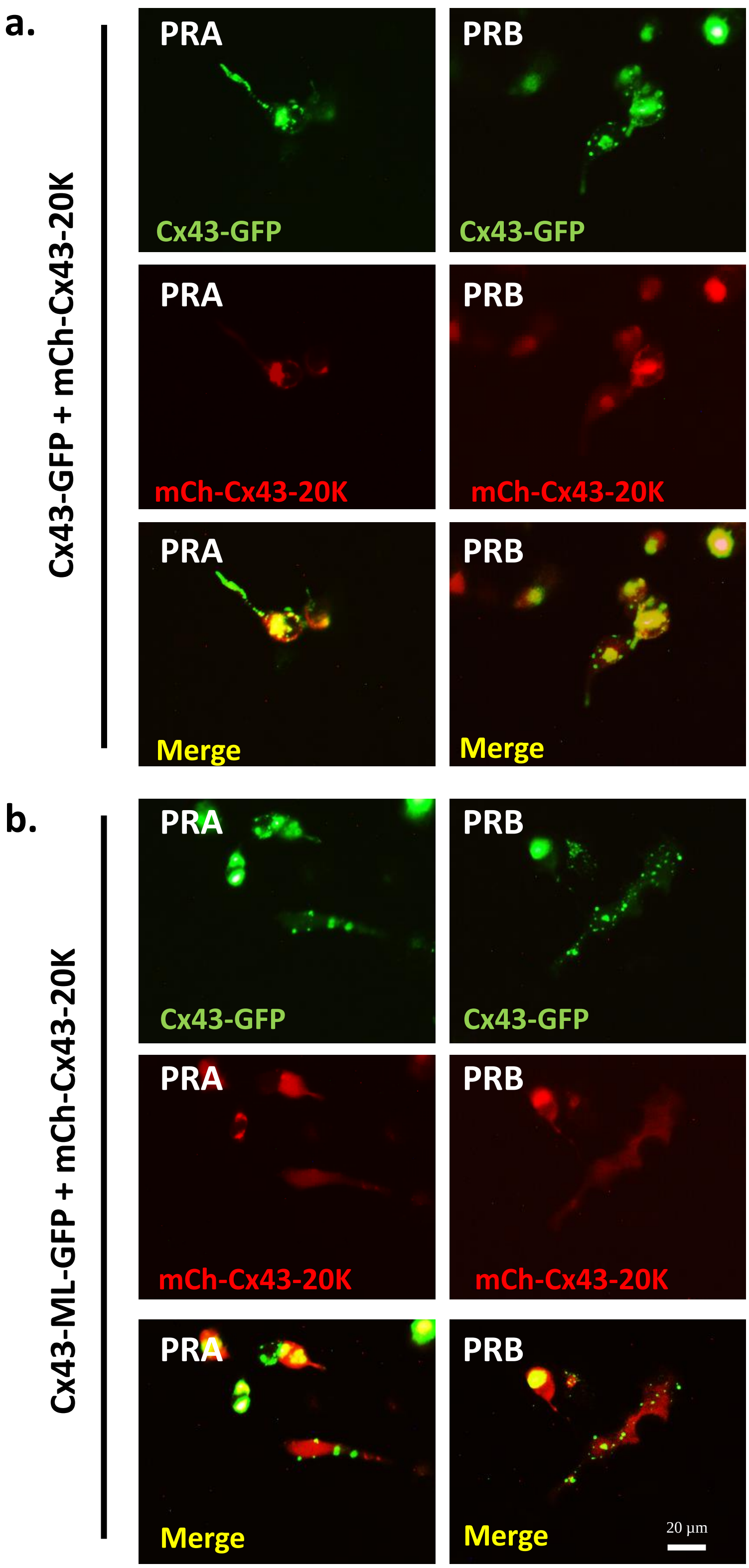

Supplementary Figure 5: Cx43-20K protein mediates the effect of PRs on Cx43 forward trafficking in HEK-293T cells. HEK-293T cells were transfected with PRA or PRB, mCh-Cx43-20K and **(a)** Cx43-GFP or **(b)** Cx43-ML-GFP plasmid constructs and treated with P4 (100 nM) for 24 h. Images show localization of total Cx43 with green fluorescence, Cx43-20K with red and their co-localization with yellow colour in merged pictures. Results show that **(a)** The expression of Cx43-20K restored Cx43 forward trafficking in PRB expressing cells and **(b)** Cx43 mutant vector (Cx43-ML-GFP) when co-expressed with Cx43-20K vector, the Cx43 forward trafficking was similarly promoted in PRA and PRB expressing cells. Scale bar = 20 µm.

a.

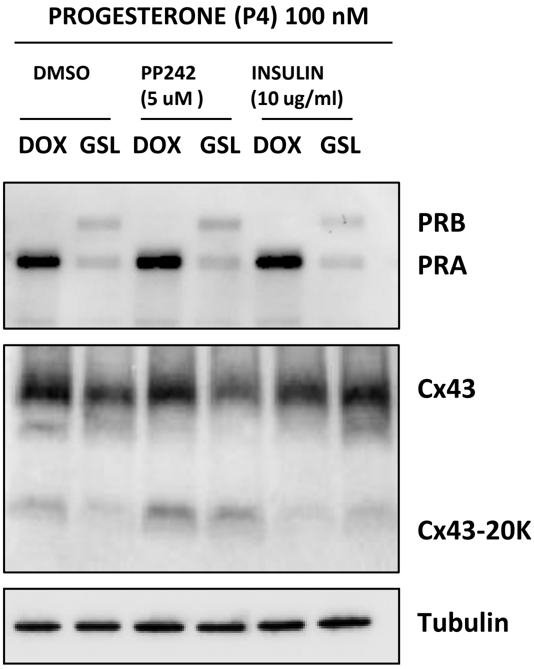

b.

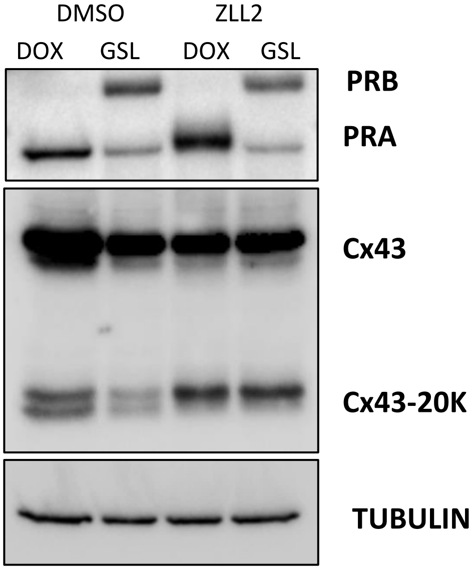

Supplementary Figure 6: Western blot validation of Cx43-20K expression. hTERT-HM$^{A/B}$ cells induced to express either PRA (with DOX) or PRB (with GSL) and stimulated with P4 (100 nM) were treated with **(a)** mTOR inhibitor (PP242, 5 µM) or mTOR stimulator (insulin, 10 µg/ml) or **(b)** gamma secretase inhibitor (ZLL2, 50 µM) for 24 h. Results show that **(a)** mTOR inhibition results in recovery of Cx43-20K in PRB expressing cells while stimulation of mTOR pathway via insulin results in downregulation of Cx43-20K expression even in the PRA expressing cells, **(b)** the expression of Cx43-20K in hTERT-HMA/B cells is not repressed by gamma secretase inhibitor suggesting that Cx43-20K isoform is not regulated via proteolytic cleavage mechanism by PRs

a.

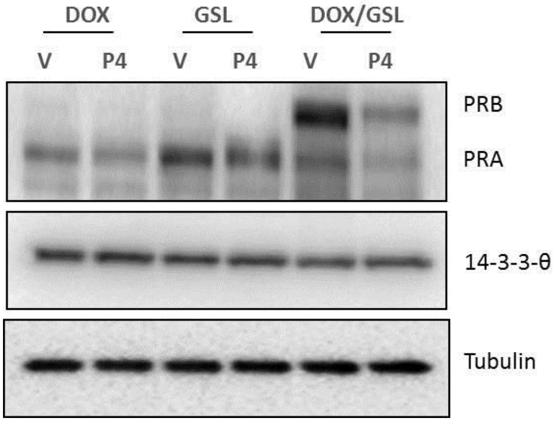

b.

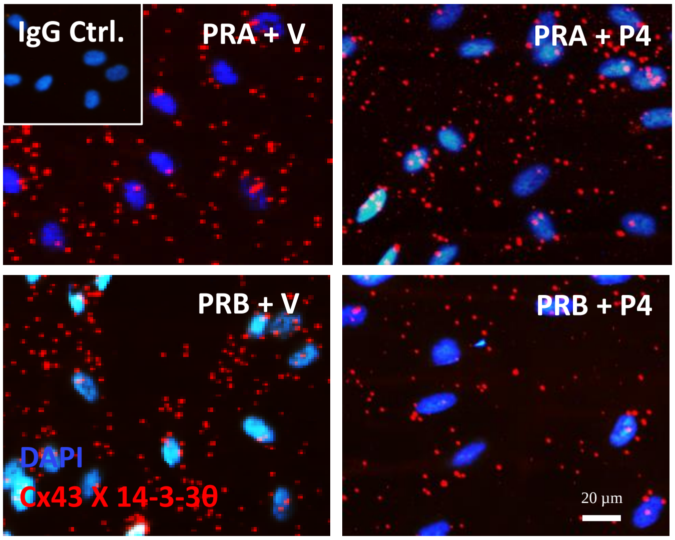

Supplementary Figure 7: hTERT-HM$^{A/B}$ cells were induced to express either PRA (with DOX) or PRB (with GSL) or both and stimulated with vehicle or P4 (100 nM) for 24 h (**a**) or 2 h (**b**). **a)** Western blot shows induction of PRs with respective treatment and expression of 14-3-3θ protein. **b)** Proximity Ligation Assay (PLA) analysis was performed to determine the interaction between Cx43-FL and 14-3-3θ protein. Nuclei were counter stained with DAPI (blue) and red fluorescence shows interaction between Cx43 and 14-3-3θ protein. Scale bar = 20 µm.

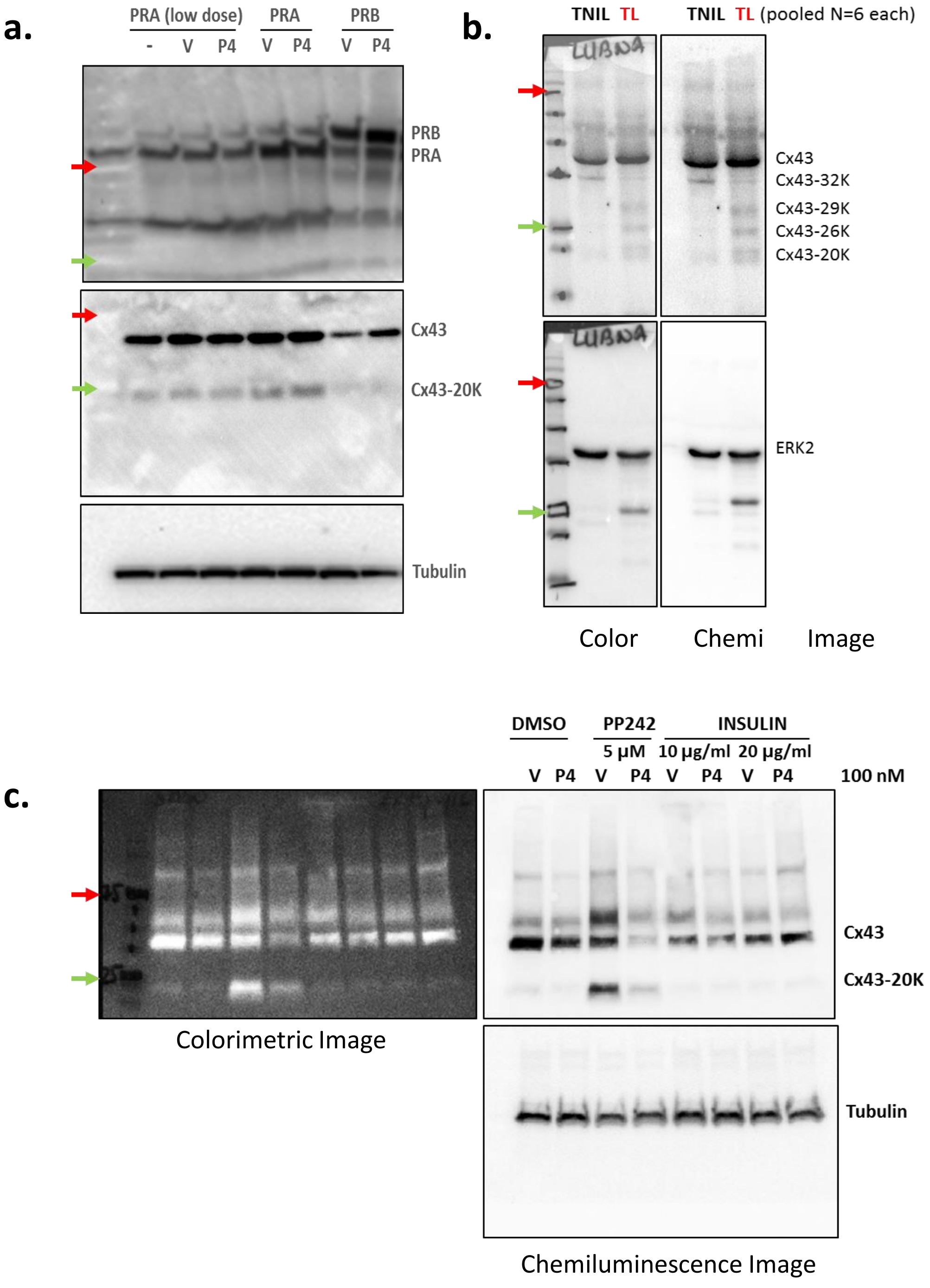

Supplementary Figure 8: Uncropped blots from Figure 3 (a, b) and Figure 5 (g).
